# CMAP Scan Examination of the First Dorsal Interosseous Muscle After Spinal Cord Injury

**DOI:** 10.1109/TNSRE.2021.3088061

**Published:** 2021-06-30

**Authors:** Ya Zong, Zhiyuan Lu, Maoqi Chen, Xiaoyan Li, Argyrios Stampas, Lianfu Deng, Ping Zhou

**Affiliations:** Department of Rehabilitation Medicine, Ruijin Hospital, Shanghai Jiao Tong University School of Medicine, Shanghai 200025, China.; Institute of Rehabilitation Engineering, University of Health and Rehabilitation Sciences, Qingdao 266024, China; Institute of Rehabilitation Engineering, University of Health and Rehabilitation Sciences, Qingdao 266024, China; Department of Bioengineering, University of Maryland, College Park, MD 20742 USA.; Department of Physical Medicine and Rehabilitation, University of Texas Health Science Center at Houston, Houston, TX 77030 USA, and also with the TIRR Memorial Hermann Research Center, Houston, TX 77030 USA.; Shanghai Key Laboratory for Prevention and Treatment of Bone and Joint Diseases, Department of Orthopaedics, Shanghai Institute of Traumatology and Orthopaedics, Ruijin Hospital, Shanghai Jiao Tong University School of Medicine, Shanghai 200025, China; Institute of Rehabilitation Engineering, University of Health and Rehabilitation Sciences, Qingdao 266024, China

**Keywords:** Compound muscle action potential (CMAP), CMAP scan, motor unit number estimation (MUNE), MScanFit, first dorsal interosseous (FDI) muscle, spinal cord injury (SCI)

## Abstract

The study assessed motor unit loss in muscles paralyzed by spinal cord injury (SCI) using a novel compound muscle action potential (CMAP) scan examination. The CMAP scan of the first dorsal interosseous (FDI) muscle was applied in tetraplegia (n = 13) and neurologically intact (n=13) subjects. MScanFit was used for estimating motor unit numbers in each subject. The D50 value of the CMAP scan was also calculated. We observed a significant decrease in both CMAP amplitude and motor unit number estimation (MUNE) in paralyzed FDI muscles, as compared with neurologically intact muscles. Across all subjects, the CMAP (negative peak) amplitude was 8.01 ± 3.97 mV for the paralyzed muscles and 16.75 ± 3.55 mV for the neurologically intact muscles (p < 0.001). The CMAP scan resulted in a MUNE of 59 ± 37 for the paralyzed muscles, much lower than 108 ± 21 for the neurologically intact muscles (p < 0.001). No significant difference in D50 was observed between the two groups (p= 0.2). For the SCI subjects, there was no significant correlation between MUNE and CMAP amplitude, or any of the clinical assessments including pinch force, grip force, the Graded Redefined Assessment of Strength, Sensibility and Prehension (GRASSP) score, and SCI duration (p > 0.05). The findings provide an evidence of motor unit loss in the FDI muscles of individuals with tetraplegia, which may contribute to weakness and other hand function deterioration. The CMAP scan offers several practical benefits compared with the traditional MUNE techniques because it is noninvasive, automated and can be performed within several minutes.

## I. INTRODUCTION

Spinal cord injury (SCI) has a detrimental effect on both short-term and long-term health-related qualit of life because damage to the spinal cord interrupts the sensory and motor pathways, resultin in varying degrees of paralysis, loss of sensation, and autonomic dysfunction. After SCI, a range of pathological changes can arise in motor neuron, motor control, and muscle properties, resulting in development of muscle spasticity, decreased force production and loss of muscle function. Skeletal muscle is the primary organ involved in the generation of force for movement and a main effector organ of impairment in SCI that results in disability. It is important to investigate changes in paralyzed skeletal muscles after SCI, which can help develop tailored rehabilitation strategies to maximize motor recovery. Of particular importance is to assess and understand complex motor unit changes after SCI because it is the final common pathway for neuromuscular control and provides a basic structure-function framework for the examination of neural and muscular disorders.

Pathological alterations in motor unit or spinal motor neuron functions following SCI have been assessed by electrophysiological studies (in addition to the known effects of spinal white matter damage on voluntary muscle activation). Using different electromyography (EMG) techniques (e.g., concentric needle EMG, single fiber EMG, macro-EMG and conventional surface EMG), investigators have reported the presence of electrophysiological abnormalities from impaired muscles. These include fibrillation and positive sharp waves [1], [2], long lasting involuntary muscle activity [3], [4], increased motor unit action potential (MUAP) size and waveform complexity [5]–[7], disorganization of motor unit control properties [8]–[10], and abnormal jitters [11], [12]. The compound muscle action potential (CMAP) measurement and motor unit number estimation (MUNE) have also revealed varying degrees of motor unit loss after SCI.

In the current study, we present a novel examination of individuals with SCI by application of the CMAP scan recordings. Recently developed, CMAP scan is a novel, noninvasive neurophysiological tool, which records the electrical activity of a muscle in response to a full spectrum of transcutaneous stimulations of the motor nerve [13]. The objective of the study was to assess and understand SCI-induced muscle alterations by comparing CMAP scan characteristics between SCI and matched healthy control subjects. Based on CMAP scan recordings, a novel MUNE method called MscanFit [14], [15] was used to examine whether the numbers of motor units in muscles impaired by SCI are lower than those estimated from matched control muscles. Assessment of motor unit loss of paralyzed muscles after SCI can help understand complicated determinants of SCI-induced muscle paresis and provide evidence of spinal motor neuron degeneration after the injury [16]–[19]. The CMAP scan used in this study is noninvasive and performed quickly with automated analyses, and therefore, has great promise for its potential applications in clinical practice.

## II. METHODS

### A. Subjects

Thirteen individuals with SCI tetraplegia participated in the study (10 males and 3 females). Thirteen neurologically intact subjects were also recruited as control group (8 males and 5 females). One subject was left-handed and the other subjects were right-handed for each of the two groups. The protocol was approved by the Protection of Human Subjects (CPHS) at University of Texas Health Science Center at Houston (UTHealth) and TIRR Memorial Hermann Hospital (Houston, TX). All participants gave written informed consent in accordance with the Declaration of Helsinki. Table I shows the SCI subject information (for the right three columns, please refer to Results section).

### B. Experiments

The first dorsal interosseous (FDI) muscle, innervated by the C8/T1 nerve roots, was examined. The skin temperature was maintained at approximately 32°C in the experiment. For the SCI group, the right hand was tested. For the control group, the test was performed on the individual’s dominant hand. For each tested hand, the grip force was measured by Jamar Plus+ Digital Hand Dynamometer (Patterson Medical, Warrenville, USA), and the pinch force was measured by PG-60 Pinch Gauges (B&L Engineering, Santa Ana, USA).

#### 1) CMAP Recording:

**Fig. 1.**
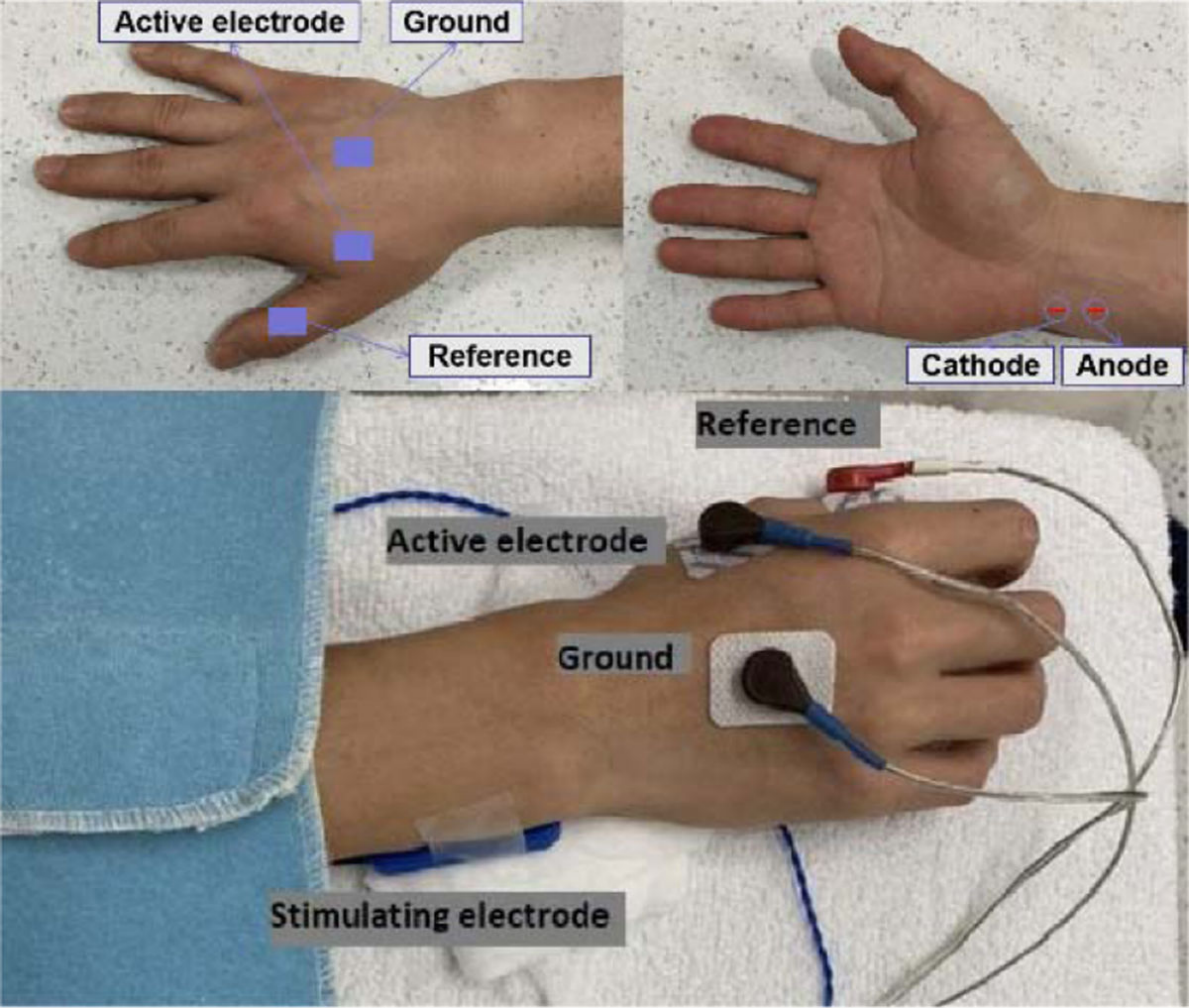
The placement of the electrodes for CMAP recording.

Alcohol pads were used to cleanse the area before the electrodes were placed. The subjects were seated comfortably in their wheelchair with shoulder and elbow flexed 90° and forearm in semi-prone position on a height-adjustable table. Figure 1 shows the electrode placement for CMAP recording, which is the summation of a group of almost simultaneous action potentials from motor units activated by electrical stimulation. The active electrode and reference electrode (Ag–AgCl disposable electrode, 10 mm in diameter) were placed on the motor point of the FDI muscle and the distal phalanx of thumb, respectively. The ground electrode was placed on the dorsal side of the hand. A standard bar electrode (each contact surface 9 mm in diameter, 20 mm apart) was placed 1–2 cm proximal to the wrist, for delivering electrical stimuli to ulnar nerve. The cathode of the electrode was positioned distally. Surgical tape and coban self-adherent wrap were used to firmly attach the bar electrode to the skin. During recording the examined hand was restrained in supination by Nylatex® wraps (4″ width) in order to minimize movement artifacts. All the data were collected using UltraPro S100 EMG system, with a 48 kHz sampling rate and a 24-bit ADC resolution (Natus Neurology Incorporated, Middleton, WI, USA).

#### 2) CMAP Scan:

A dedicated CMAP scan program (developed and installed on the UltraPro S100 EMG system by Natus Neurology) was used to record the progressive recruitment of motor units in response to repetitive transcutaneous stimulation of motor nerve. Preceding CMAP scan, there was an automatic search for electrical stimulation intensities S0 and S100 (measured in mA) which is defined as stimulation intensities required to elicit lowest threshold motor unit and maximum CMAP amplitude respectively. Following S0 and S100 determination, stimulation intensities were adjusted to cover the entire recruitment range. Then the CMAP scan was started. Throughout the scan, the (monophasic) stimulus was delivered at a 0.1 ms pulse duration, 500 stimulus number, and stimulus frequency of 2 Hz with the intensity declining linearly within the range. It took less than 5 minutes to complete the scan.

### C. Data Analysis

#### 1) D50 Calculation:

The CMAP amplitude which is defined as the difference between baseline to negative peak of the waveform, and corresponding stimulus intensities were extracted for generating the stimulus-response curve. From each scan, the number of largest consecutive differences that are needed to build-up 50% of the maximum CMAP, called D50, was calculated [20]. This index has proved to a useful parameter for quantifying CMAP scan discontinuities.

To determine D50, as described in [20], the *N* recorded CMAP amplitudes are expressed as percentage of the maximum CMAP and ranked by size,

$$A=\left[A_{1},A_{2},\cdots A_{\text{n}},\cdots A_{N}\right],\;A_{1}\leq A_{2}\leq \cdots A_{n}\cdots A_{N},$$

where *N* = 500. Then, between these ranked CMAP amplitudes, the consecutive differences are calculated:

$$\text{Δ}A=\left[\text{Δ}A_{1},\text{Δ}A_{2},\cdots \text{Δ}A_{n},\cdots \text{Δ}A_{N-1}\right],$$

where $\text{Δ}A_{n}=A_{n+1}-A_{n}$. Next, these consecutive differences are ranked again from largest to smallest, and their cumulative sum is calculated:

$$M=\left[M_{1},M_{2},\cdots M_{n},\cdots M_{N-}\right],$$

where $M_{1}\leq M_{2}\leq \cdots M_{n}\cdots \leq M_{N-1}$. Here *M*_1_ equals the size of the largest consecutive difference present in the CMAP scan (expressed as percentage of the maximum CMAP), *M*_2_ is the sum of the largest and second largest consecutive differences, and so on, and $M_{N-1}=100\text{\%}$.

Therefore, ***M*** is the array of the cumulatively summed ranked consecutive differences in the CMAP scan. To examine the largest consecutive differences (i.e., those elements of ***M*** with low sample number *n*), a threshold is set at 50% of the maximum CMAP. D50 value is determined as the smallest value of *n* at which ***M*** exceeds this threshold [28]. For each of the examined muscles, D50 value of the CMAP scan was calculated, and compared between paralyzed and matched control muscles.

#### 2) MScanFit:

MScanFit, a program developed by Bostock [14], was used in this study for estimating motor units number based on a muscle’s CMAP scan data. Unlike Bayesian MUNE that needs substantial time to complete [21], [22], MScanFit is an automatic and a quick method for estimation of motor unit number, which applies a mathematical model to simulate the recorded CMAP scan. As described in [14], [15], the modelled CMAP scan is adjusted sequentially until its discrepancy from the experimental CMAP scan can be minimized, i.e. the modelled CMAP scan is best ‘fitted’ to the recorded one. These data were then imported to MScanFit program for MUNE calculation. Default setting (parameter assignment) of the program was used for the calculation. For each CMAP scan, the MScanFit program was performed three times, and the resultant MUNE value with the minimum percentage error <7% was accepted for further analysis [14]. If all the three percentage errors were greater than 7%, the program was performed again until the percentage error was less than 7%.

### D. Statistical Analysis

Normality of the data was evaluated using Kolmogorov-Smirnov test. Independent-samples t-test (for normal distribution) or Mann-Whitney U test (for non-normal distribution) was performed to examine whether significant differences existed in MUNE, CMAP amplitude, D50, and stimulus intensity parameters between the SCI and the control groups. Pearson test was performed to examine whether there was a correlation among MUNE and CMAP amplitude, pinch force, grip force, GRASSP score, and SCI duration. Data are presented as mean ± standard deviation for normal distribution, and median (minimum - maximum) for non-normal distribution. Statistical significance was set as p < 0.05.

## III. RESULTS

CMAP scan of the FDI muscle was successfully recorded from each SCI or healthy control subject. The stimulus intensity parameters used to generate each scan were obtained, including S0, S50, S100 and the stimulus range (S0 to S100).

As Table II shows, S0 was significantly higher for the SCI group than the control group (p = 0.01). There was no significant difference in S50, S100 and stimulating intensity range between the SCI and control subjects (p = 0.19, 0.16, 0.98, respectively) (Table II).

**Fig. 2.**
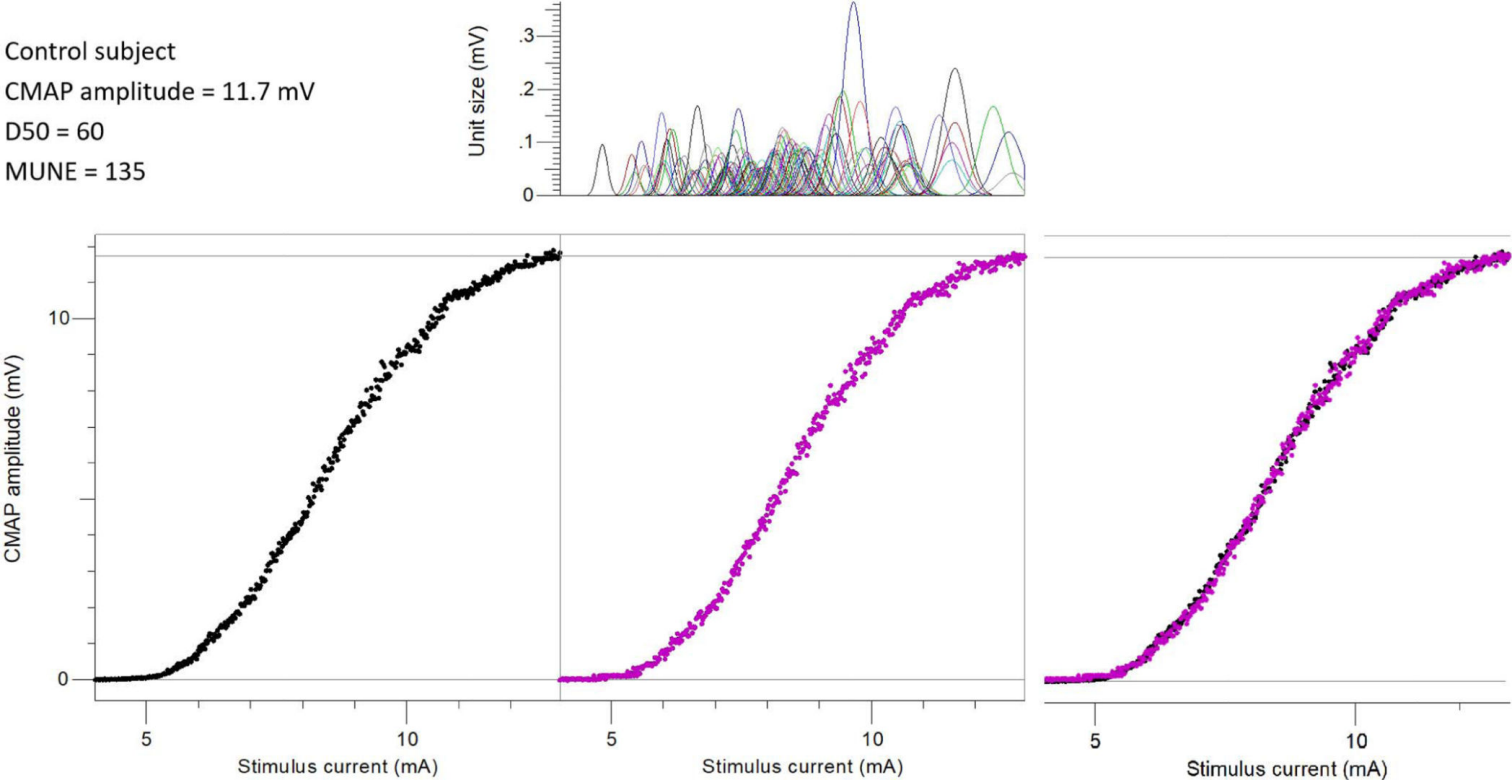
Bottom left: an example of the experimental CMAP scan from a healthy control subject; Bottom middle: the modeled CMAP scan using the MScanFit program; Bottom right: The overlap of the experimental and modeled CMAP scans; Top: the estimated motor unit action potential distribution.

**Fig. 3.**
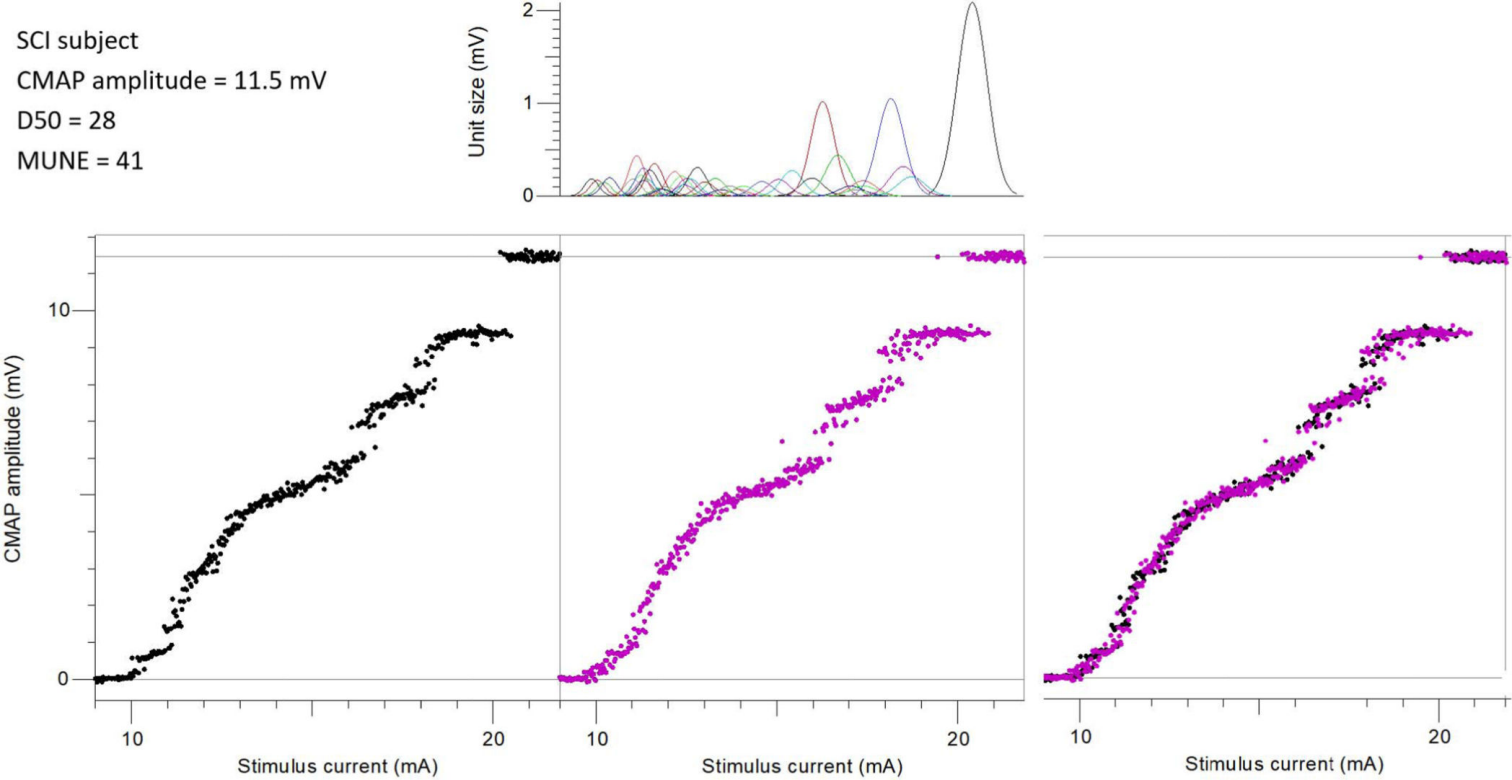
Bottom left: an example of the experimental CMAP scan from an SCI subject; Bottom middle: the modeled CMAP scan using the MScanFit program; Bottom right: The overlap of the experimental and modeled CMAP scans; Top right: the estimated motor unit action potential distribution. (Note: This is subject S08 in Table I, where the CMAP amplitude was recorded from the nerve conduction velocity test).

Figure 2 and Figure 3 show an example of the CMAP scan recorded from a healthy control subject and a SCI subject (S08), respectively. It was observed visually that the SCI subject’s CMAP scan curve had obvious gaps while the control subject’s CMAP scan was contiguous and smoother. The estimated MUAP distribution and the fitted CMAP scan from the MScanFit program are also shown in Figures 2 and 3. It indicates that the D50 values (60 vs 28) and the motor unit number (135 vs 41) estimated from the two CMAP scans were dramatically different although the CMAP amplitude (11.7 mV vs 11.5 mV) of the two subjects was very similar.

As shown in Table III, the maximum CMAP amplitude was 8.01 ± 3.97 mV for the SCI subjects and 16.75 ± 3.55 mV for the control group (p < 0.001). The D50 of the CMAP scan was 37 ± 17 for the SCI group, and 44 ± 9 for the control group, respectively. There was no significant difference in D50 between the two groups (p = 0.2) (Table III). The average MUNE of the FDI muscle derived from the MScanFit was 59 ± 37 for the SCI subjects, which was significantly lower than 108 ± 21, derived from the control group (p < 0.001).

The pinch force, grip force, GRASSP score and SCI duration were 2.7 ± 3.5 kg, 8.3 ± 12.7 kg, 57.2 ± 36.2, and 9.5 ± 9.2 years for the SCI subjects. The measures for each individual SCI subject are presented in Table I, together with CMAP, MUNE and D50 values. No significant correlation was found between MUNE and CMAP amplitude, or any of the clinical assessments (pinch force, grip force, GRASSP score, SCI duration) for the SCI subjects (p > 0.05). The MUNE from SCI subjects tended to negatively correlate with the duration of injury, although no significance was observed (r = −0.5, p = 0.08).

## IV. DISCUSSION

This study presents a novel examination of affected FDI muscle after SCI based on CMAP scan recording, which offers several practical benefits compared with the traditional MUNE techniques. Various MUNE techniques (such as incremental stimulation based MUNE, multipoint stimulation MUNE, decomposition or spike triggered averaging based MUNE, statistical MUNE, F wave-based MUNE, high density surface EMG based MUNE, Bayesian MUNE, etc.) have been developed [23], [24]. Some of these techniques have been applied to examine motor unit alterations after SCI [16]–[19]. The MUNE methods are usually affected by a number of factors including the sample size of motor units and the MUAP amplitude distribution [23], [24]. For example, incremental stimulation MUNE is determined by the motor unit recruitment preference in response to electrical stimulations; most statistical MUNE methods depend on responses at only a few stimulus levels; spike-triggered averaging or surface EMG decomposition-based methods depend primarily on motor units recruited at low levels of voluntary effort [25], [26]. The Bayesian MUNE attempts to provide an estimate of all the motor units in a muscle, but it requires very complex computations, available only in a limited number of centers and can take several hours to complete, even with a high-powered computer [21], [22]. Compared with most previous methods, CMAP scan based MUNE has advantages of providing information about all the motor units contributing to the CMAP [13]. By fitting a model to an experimental CMAP scan curve, the inherent bias of extrapolating from a small sample of motor units can be avoided [14], [15]. Compared with Bayesian MUNE, the data processing of CMAP scan is straightforward and much easier to implement. CMAP scan protocol is noninvasive, and can be performed automatically and quickly (within several minutes), making it a clinically applicable tool for examining or tracking neuromuscular disorders. Compared with another clinically applicable MUNE method, motor unit number index (MUNIX) [27], CMAP scan based MUNE can be performed for those patients who are not able to generate voluntary muscle contraction, while MUNIX requires recording of voluntary surface EMG at different contraction levels (from minimum to maximum) [28].

CMAP scan MUNE has been tested in several previous studies in both healthy control subjects and individuals with neuromuscular diseases (such as amyotrophic lateral sclerosis), and has shown excellent intra- and inter-rater reproducibility [34]–[38]. In the present study, the same CMAP scan protocol (stimulus steps, duration, frequency, etc.) was used for both SCI and the healthy control subjects to ensure that the comparison in the derived MUNE or D50 values between the two groups was not compromised by the difference induced from different CMAP scan protocols [29]. In line with previous observations [2], [30]–[32], our results confirmed that the CMAP amplitude of the FDI muscle was significantly lower in SCI subjects than in the healthy control subjects. Several previous MUNE studies of individuals with SCI have reported variable levels of motor unit loss in paralyzed muscles [16]–[19]. Our results also revealed a significant reduction of motor unit numbers after SCI. Of particular note, we found that some SCI subjects’ CMAP amplitudes were within normal range but the estimated motor unit number was significantly reduced, due to compensatory muscle fiber reinnervation. This observation is demonstrated in Figure 2 and Figure 3, where the number of motor units was dramatically different even the maximal CMAP amplitude of the two subjects was almost the same. This suggests that the MScanFit MUNE was a more sensitive parameter than CMAP amplitude for assessment of motor unit loss in the case of muscle fiber reinnervation.

Sleutjes *et al.* reported that D50 was a potentially useful index to monitor neurogenic disorders with moderate to severe motor unit loss [20]. This index is related to decreases in motor unit number and increases in motor unit size. The average D50 was reported to be 39 for the healthy subjects in the previous study [20]. In our study, the average D50 was 37 ± 17 for the SCI group and 44 ± 9 for the control group, respectively. There was no significant difference between the two groups (p = 0.2). The mean D50 in SCI patients was also similar to the healthy subjects in the Sleutjes et al study [20]. In contrast, in the current study a significant difference in MUNE between two groups was observed (p < 0.001). Given that a decreased D50 is related to significant motor unit loss, the MScanFit MUNE can be a more sensitive assessment of mild to moderate motor unit loss.

There was a significant difference in the lowest stimulus intensity (S0) between the two groups (p = 0.01), while no significant difference in S50, S100 and stimulus intensity range (S0 to S100) was observed (Table II). It remains unclear why S0 was elevated in the SCI group, which is likely related to poor sweat response and skin circulation below the injury level as a constellation of autonomic dysreflexia [39]. The initial threshold might be altered by the impedance of the skin.

The FDI muscle examined in this study is the only muscle that abducts the index finger whose nerve supply is easily accessible for clinical EMG testing. Although the CMAP amplitude and MUNE of the FDI muscle were significantly lower in SCI subjects compared with the healthy control subjects, we did not find that they were correlated to any of the clinical assessment. This is not surprising given the fact that the electrophysiological parameters in this study were estimated only from FDI, while the clinical or functional assessments involve coordination of multiple muscles. For example, besides distal hand muscles, maximum grip strength includes contributions from proximal muscles in the arm, which were not considered in the current study. In addition to motor unit number, other complex neural and muscular changes may be present in different degrees after SCI. This will also compromise the correction between MUNE and the clinical assessment.

In a previous study, Yang et al reported that some SCI patients showed a normal number of motor units long after the injury [16]. However, in the current study, although there was no significant correlation between MUNE and SCI duration, our results indicate a trend of higher MUNE for those subjects with short duration and lower MUNE for those with long duration. A larger sample size is required to test whether a significant correlation can be observed. A longitudinal tracking of MUNE in the same acute SCI patients will provide most useful information on how motor unit number changes with time after SCI.

## V. CONCLUSION

This study presents a novel application of the CMAP scan technique (which is convenient to implement and has a great potential for clinical use) in examination of muscles paralyzed by a spinal cord injury. The present cross-sectional study found a significant decrease in both CMAP amplitude and MUNE in paralyzed FDI muscles, as compared with neurologically intact muscles. The findings provide further electrophysiological evidence of motor unit loss or motor neuron degeneration following an SCI, which contributes to weakness and other hand function deterioration. This would drive treatment strategies directed to develop ways to prevent motor unit loss, such as through drug interventions and neuromodulation techniques, during the acute phase of SCI. This may also help to better understand and predict the extent of unavoidable motor unit loss and guide exercise and electric stimulation therapy to enhance the natural remyelination process. Thus, the findings have potential clinical value for the prognosis and treatment of SCI, and the evaluation of the effects of medication or therapies.

## Figures and Tables

**TABLE I:** SCI SUBJECT INFORMATION

Subject Index	Gender	Dominant Hand	Neurologie Level	ASIA	Age (years)	Years of Injury	GRASSP	Grip (kg)	Pinch (kg)	MUNE	CMAP (mV)	D50

S01	M	R	C7	B	57	24	70	0.9	0	28	4.23	17
S02	M	R	C5	D	62	10	84	15.2	4.5	42	9.21	52
S03	M	R	C6	B	21	3	34	0	0	75	8.80	56
S04	F	R	C5	C	57	1	57	2.3	1.8	141	8.46	44
S05	F	R	C5	B	39	16	31	0	0	30	4.64	32
S06	M	R	C5	D	24	1	108	45.4	11.3	74	18.04	38
S07	M	R	C4	C	53	30	90	15.4	5.0	35	5.51	35
S08	M	L	C4	D	59	3	83	19.5	8.2	41	11.81	28
S09	M	R	C1	A	21	1	0	0	0	107	8.37	71
S10	M	R	C6	C	45	4	79	0	1.4	5	1.78	2
S11	M	R	C3	C	35	15	7	0	0	76	8.38	39
S12	F	R	C3	C	54	2	4	0	0	75	6.94	43
S13	M	R	C3	D	37	13	96	9.8	3.0	43	8.00	27

AIS: American Spinal Injury Association (ASIA) Impairment Scale

GRASSP: The Graded Redefined Assessment of Strength, Sensibility and Prehension (GRASSP) Test [33]

**TABLE II:** STIMULUS INTENSITY PARAMETERS OF THE SCI AND CONTROL GROUPS

Group	S0*(mA)	S50**(mA)	S100**(mA)	Intensity range* (mA)

SCI	8.5 (4.5–14)	13.75 ± 4.20	19.38 ± 6.68	9.0 (5.0–23.0)
Control	4.5 (3.0–9.5)	11.64 ± 3.71	16.00 ± 4.95	9.5 (6.0–16.0)

	p = 0.01	t=1.358, p = 0.19	t= 1.468, p = 0.16	p = 0.98

* Mann-Whitney U test,

** Independent-samples t-test

**TABLE III:** MUNE, CMAP AMPLITUDE AND D50 VALUES OF THE SCI AND HEALTHY CONTROL GROUPS

Group	MUNE	CMAP (mV)	D50

SCI	59 ± 37	8.01 ± 3.97	37 ± 17
Control	108 ± 21	16.75 ± 3.55	44 ± 9

	t = 4.135, p < 0.001	t = 5.91 , p < 0.001	t = 1.335 , p = 0.20
